# The influence of signs of social class on compassionate responses to people in need

**DOI:** 10.3389/fpsyg.2022.936170

**Published:** 2022-08-25

**Authors:** Bennett Callaghan, Quinton M. Delgadillo, Michael W. Kraus

**Affiliations:** ^1^The Graduate Center, City University of New York, New York, NY, United States; ^2^Columbia Business School, Columbia University, New York, NY, United States; ^3^School of Management, Yale University, New Haven, CT, United States; ^4^Department of Psychology, Yale University, New Haven, CT, United States

**Keywords:** compassion, emotion, social class, socioeconomic status, economic inequality, person perception, intergroup relations, prosocial behavior

## Abstract

A field experiment (*N* = 4,536) examined how signs of social class influence compassionate responses to those in need. Pedestrians in two major cities in the United States were exposed to a confederate wearing symbols of relatively high or low social class who was requesting money to help the homeless. Compassionate responding was assessed by measuring the donation amount of the pedestrians walking past the target. Pedestrians gave more than twice (2.55 times) as much money to the confederate wearing higher-class symbols than they did to the one wearing lower-class symbols. A follow-up study (*N* = 504) exposed participants to images of the target wearing the same higher- or lower-class symbols and examined the antecedents of compassionate responding. Consistent with theorizing, higher-class symbols elicited perceptions of elevated competence, trustworthiness, similarity to the self, and perceived humanity compared to lower-class symbols. These results indicate that visible signs of social class influence judgments of others’ traits and attributes, as well as in decisions to respond compassionately to the needs of those who are suffering.

## Introduction

Individuals from various species signal their social status with non-verbal behaviors and social symbols. These status symbols assist them in avoiding costly aggressive encounters, and they signal the availability of resources and opportunities that facilitate thriving within groups (Krebs et al., 1993; Zeil and Hofmann, 2001). Generally speaking, a relative lack of opportunity, shorter life spans, and chronic stress accompany low status in various species, humans included (Sapolsky, 2004). Research in the social sciences also suggests that perceivers across the globe judge low-status individuals–especially those from denigrated groups, such as those experiencing poverty and homelessness–in negative terms: as low in warmth (untrustworthy) and competence (incapable; Cuddy et al., 2002, 2008), as lacking traits typically associated with humanity and personhood and having traits associated with animality (Laughnan et al., 2014), and as possessing inferior genes (Kraus and Keltner, 2013). These types of perceptions motivate avoidance and ostracism directed toward lower-status groups and individuals (e.g., Bastian and Haslam, 2010). In the present research, we examined how visible symbols of status (in particular, those that communicate one’s social class position in society; Kraus et al., 2009) influence compassionate responding in contexts of suffering and need.

Compassion is a complex prosocial emotion defined as concern for the suffering of others and the motivation to help ease that suffering (e.g., Goetz et al., 2010; Gilbert, 2017; Mascaro et al., 2020). Related to sympathy, empathy, and empathic concern, compassion is uniquely positioned as an affective state that tracks with the concern for reducing the suffering of another (Ekman, 1992; Nussbaum, 1996), and the presence of suffering is required to define prosocial or altruistic behavior as compassionate responding (Batson et al., 1989; Cialdini et al., 1997; Oveis et al., 2010).

Critically, however, theoretical analyses of compassion’s origins posit that deservingness (broadly defined), combined with suffering, is central to compassionate responding (Goetz et al., 2010; Oveis et al., 2010). For instance, evolutionary accounts of compassion are rooted in theorizing on reciprocal altruism. Altruism is defined as selfless behavior that may or may not represent compassionate behavior, which requires acknowledgment of suffering; however, altruistic behavior is the primary expression of compassion (e.g., Goetz et al., 2010). These accounts of reciprocal altruism hinge on the assumption that altruists will choose to benefit those perceived as altruistic themselves (Trivers, 1971; Frank, 1988; Henrich, 2004), other kin (Hamilton, 1964), or others who are deemed trustworthy. Theoretically, from an evolutionary perspective, communities can most efficiently leverage the benefits of reciprocal altruism and cooperation if prosocial individuals tend to help other prosocial individuals and avoid those who might take advantage of or squander their kindness, such as dishonest individuals who feign suffering. For similar reasons, according to these accounts of compassion, those who are seen as responsible for their own suffering in the first place (and may thus be seen as blameworthy for their plight) are also seen as less deserving of help (Axelrod and Hamilton, 1981; Goetz et al., 2010). Overall, then, individuals tend to help others during their times of suffering and need based on whether those others are seen as “deserving”: as genuinely suffering, not responsible for their suffering, and generally trustworthy and prosocial themselves (and thus likely to help others in the future, Goetz et al., 2010).

Our expectation that symbols of social class will influence compassionate responses synthesizes these prior theoretical accounts of the deservingness appraisals that precede compassion (Goetz et al., 2010) with several lines of evidence suggesting that others’ social class (i.e., one’s socioeconomic position in society, generally assessed in terms of education, income, and occupational status; Adler et al., 1994) can be gleaned from the recognition of status symbols. Consistent with theories of social comparison (Festinger, 1954), individuals are motivated to compare their economic standing to that of others in order to form opinions about their own performance and abilities in social domains; they are so motivated, in fact, that humans engage in social comparison even when it results in negative feelings of relative deprivation and perceptions of having reduced resources (e.g., Buunk et al., 2003). With respect to social class, these comparisons occur across a number of contexts, rapidly, and with little input. For instance, research on person perception reveals that individuals perceive social class with accuracy based on 60 s interactions with strangers (Kraus et al., 2009), photographs posted on social media (Becker et al., 2017), and pronunciation in brief speech (Giles and Sassoon, 1983; Labov, 2006; Kraus et al., 2019).

Perceptions of social class derived from such status symbols, in turn, inform the social perception and judgment of strangers. Prior research suggests that visual depictions of poverty can elicit perceptions of low warmth and competence in a given target (Harris and Fiske, 2009) and facilitate processes of alienation and dehumanization. These same visual depictions, for instance, also elicit perceptions that targets are dissimilar to the self (Harris and Fiske, 2009), and stereotypes of various lower-class social groups characterize their members as animalistic and lacking distinctly human qualities (Laughnan et al., 2014). Congruently, regions of the brain associated with person perception show less activation when middle-class perceivers view poor or homeless targets, as compared to middle-class ones (Harris and Fiske, 2006). Thus, the ability to perceive social class in others not only allows humans to identify social hierarchies–and their own place within them–but it also allows for patterns of social perception that implicitly justify these hierarchies, portraying those at the bottom as incompetent or undeserving.

As a result, these status-linked patterns of social perception may often direct compassion toward those exhibiting symbols of higher, compared to lower, social class. Several lines of research indirectly support this contention. Little prior research has directly and explicitly investigated the influence of status symbols on compassionate responding, though some research has investigated status signaling (or similar concepts) and its relationship to behaviors that might be considered compassionate responding or that represent constructs that are similar to compassionate responding in that they are other-focused and involve either placing trust in others (e.g., cooperation) or investing time or resources into others’ wellbeing (e.g., prosocial behavior, helping behavior). For instance, people prefer to cooperate with individuals who are perceived to be both warm and competent (e.g., Anderson and Kilduff, 2012), and they exhibit contempt–rather than compassion–for those who appear to lack both these qualities (Cuddy et al., 2008; Goetz et al., 2010). Studies also suggest that individuals experience more compassion toward the suffering of others who are more, rather than less, similar to the self (Cialdini et al., 1997; Oveis et al., 2010). Finally, dehumanization processes elicit judgments that targets are less worthy of moral consideration (e.g., Bandura, 2002) and, therefore, compassion (Fiske, 2009; Goetz et al., 2010). As noted, each of these differential patterns of perception can be elicited by observable social class signals.

A smattering of early research has also investigated the relationship between perceived status (measured in a variety of implicit and explicit ways) and outcomes similar to compassionate responding. Using the “wrong number” technique (Gaertner and Bickman, 1971), Goodman and Gareis (1993) found that individuals were less likely to place a phone call on behalf of confederates who stated they had a low-status occupation (i.e., gas station attendant) as opposed to a high-status occupation (i.e., lawyer) or an unspecified occupation. Similarly, in the context of assessing donation behavior at an Indian university, Pandey (1979) found that professors (a high-status role) who had identified themselves as such were more successful at eliciting donations for victims of a recent flood than student counterparts. In another study, women were more likely to receive help packing a dropped bag of groceries when the make and model of their cars reflected high, rather than low, status (Solomon and Herman, 1977). In these instances, status was not manipulated through the use of visible status signals and the status in question does not necessarily reflect socioeconomic status (SES). However, this prior research does suggest that those of higher status (variously defined) often receive more aid than those of lower-status, whether in the form of help (e.g., by receiving a favor) or money.

Other evidence for visible status symbols, in particular, influencing behavior similar to compassionate responding (i.e., costly behavior that benefits others) comes from research into analogous behaviors of ceding resources or engaging in cooperation. Bickman (1971), for instance, found that individuals were more likely to return a dime left in a phone booth to confederates dressed in upper social class sartorial symbols (i.e., business attire) as opposed to low status ones. In a more recent experiment involving a negotiation game, the largest differences in monetary concessions emerged between targets manipulated to wear similar symbols (i.e., a business suit purchased at Macy’s) and perceivers wearing their own clothing, with perceivers tending to make concessions to counterparts signaling higher social class (Kraus and Mendes, 2014). In another set of experiments, participants who received a greater initial endowment with which to play repeated rounds of a cooperative economic game tended to exacerbate initial inequalities by cooperating exclusively with other “wealthy” players–but only when these inequalities were visible (Nishi et al., 2015).

Taken together, these lines of research suggest that observable symbols of heightened social class influence the help or resources one decides to concede to or share with others. By extension, the expression of upper social class symbols–perhaps particularly if they match those expressed by perceivers (cf., Pandey, 1979; Goodman and Gareis, 1993)–might elicit more compassionate responding, especially when combined with suffering and need on the part of the signaler.

Though the above indicates conditions where high status symbols elicit preferential treatment, there are certainly conditions where people demonstrate other-focused behavior that is at least similar to compassionate responding toward those lower, rather than higher, in status. For instance, past research has found that knowledge of an individual’s relatively lower status–combined with lay conceptions of fairness, which dictate helping those most in need (Adams, 1963; Tyler, 2012)–can elicit increased prosocial behavior in the absence of clear suffering (e.g., Van Doesum et al., 2017), a tendency that represents a commonly used metric of compassion when undertaken in the presence of suffering (Goetz et al., 2010). Similarly, an analysis of donations given through the website Kiva.org (a micro-lending service designed to generate capital for small businesses in developing countries) also found that requesters who adopted expansive postures (a cross-culturally recognized signal of high status and pride) received less in the way of eventual donations (Tracy et al., 2018). Thus, signals of need communicated by lower social status might potentially outweigh countervailing signals communicated by higher social status under certain circumstances. The contexts investigated in this prior research, however, are impersonal (taking place in online settings) and may thus allow potential helpers to rely more on reasoned cognitive processes or normative expectations–processes that might not necessarily hold sway in contexts where individuals need to respond rapidly in an interpersonal context. Moreover, this prior research was not designed to investigate responses to need or suffering specifically. Research showing a predilection toward helping lower-status targets has often investigated helping behavior toward targets, in the absence of need or suffering (e.g., Van Doesum et al., 2017) or, more generally, where help was requested for a variety of specific reasons across a number of different contexts (Tracy et al., 2018).

In the current research, we investigate how compassionate responses are influenced by status signaling, and we do so in the context of aiding those suffering from homelessness. Prior research provides a precedent for using exposure to homelessness as a context for eliciting compassion that still allows for significant variability in responding: compassion toward such individuals, for instance, is contingent on feelings of empathic concern for Batson et al. (1989) or self-other overlap with (Cialdini et al., 1997) targets, which can differ from one perceiver to the next. The particulars of the situation can also shape compassionate responding. Even when normative prescripts that dictate helping those in need are made salient, individuals often overlook the suffering of unhoused individuals–especially in the face of competing demands (Darley and Batson, 1973). Instead of leveraging variation in individual differences or the experimental context, the current research manipulates status signals emitted by individuals themselves, while maintaining this context of need and suffering. Theoretically, these signals guide inferences of warmth, competence, similarity, and humanity, which determine whether those making such inferences see individuals as deserving of compassion in the first place. We hypothesized that observable symbols of high, relative to low, social class would elicit increased behavior indicative of compassionate responding on the part of perceivers.

We tested our hypothesis in a field study in urban areas situated in two large metropolitan cities in the United States. A confederate solicited donations for the homeless while wearing symbols of lower or upper social class on consecutive days. This context–of soliciting donations on public streets–is likely to elicit perceptions of suffering and low baseline social class regardless of the manipulation of status signaling, but our central prediction was informed by expectations that higher social class symbols in this context would elicit greater compassion due to heightened perceptions of the confederate’s warmth, competence, humanity, and similarity to the self. We then assessed whether targets based on this confederate differentially elicited these same patterns of social perception in a follow-up experiment. Notably, and especially given past research on the influence of status perceptions on behavior in related domains of prosocial (Van Doesum et al., 2017) and altruistic (Tracy et al., 2018) behavior–which typically find that lower perceived status is associated with higher perceived need and greater generosity–support for our hypotheses in the current study would suggest that, ironically, there are contexts in which those who signal lower status (and thus might need help more or be perceived as in greater need) actually benefit less from the compassion of others. Data, analyses, and materials used in both experiments are available at https://osf.io/bxw7g/?view_only=7d6eac8f51cd4a819e829ba386fdcf46.

For reasons detailed below (see Section “Procedure”), we avoided intentionally misleading participants by telling them that the donations were for the confederate himself or that the confederate was unhoused. Given the brief nature of these interactions and the relatively commonplace occurrence of panhandlers within urban areas, we assume that most participants in this study approached this situation in much the same manner they would approach other such individuals soliciting money–in which case, donations would presumably be given to the ostensibly unhoused confederate, under the understanding that the confederate himself would keep them (i.e., the confederate was seen as the ultimate recipient of compassionate responding). In such an instance, the observed behavior of donating to the confederate, on the part of passersby, maps straightforwardly onto accepted definitions of compassion within the literature, as a “feeling that arises in witnessing another’s suffering and that motivates a subsequent desire to help” (Goetz et al., 2010, p. 352).

However, we acknowledge that not all individuals may have perceived the confederate in this manner and that there may be differences, based on condition, in tendencies to perceive the confederate otherwise: for instance, participants may have inferred that the funds would be sent elsewhere to third-parties suffering from homelessness or to charities aimed at alleviating such suffering, making the confederate a facilitator of compassionate responding rather than a direct recipient. In this case, the confederate may have been viewed as trustee for the donated funds or a stand-in for those suffering from poverty and homelessness generally.

For this reason, we refer specifically to contexts of poverty and homelessness that activate perceptions of need and suffering rather than assume that participants perceive the confederate himself as experiencing said need and suffering. Likewise, our hypotheses are constrained to refer to status signaling within that same context, broadly defined. In other words, the only assumption we adopt with respect to donation behavior on the part of participants is that donations given were intended to alleviate suffering stemming from poverty and homelessness. Such a definition still reflects compassionate responding, regardless of how the confederate was viewed with respect to such responding (i.e., as recipient or facilitator): it is appropriate, at least, insofar as one might define donations given after watching an advertisement for a charity as compassionate responding–even if donors likely know that donations would not benefit the precise individuals pictured suffering in such an advertisement–because such exposure “motivates a subsequent desire to help” within a specific context. By necessity, however, we adopt a somewhat looser definition than those that require “witnessing another’s suffering,” strictly speaking. Therefore, we acknowledge that while the suffering to which participants responded is defined and circumscribed by the experimental context, it may not be perceived as the suffering of the confederate himself. Moreover, while we operationalize donations to the confederate as compassionate behavior regardless of participants’ interpretation of the situation, the precise manner in which participants enacted compassion and construed their behavior toward the confederate (e.g., as helping him or placing their trust in him to help others) may have differed across conditions.

As is the case in everyday instances of those responding to individuals soliciting money on the street (even outside of a research context), individual donors may have perceived the confederate and the impact of their own donations in a multitude of ways, the full extent of which are impossible to know. Thus, we can only draw firm conclusions about the influence of status signaling on compassionate responding within contexts of need and suffering (rather than conclude that donors were attempting to alleviate the pain and suffering of a specific individual). While our predictions are informed primarily–though not exclusively (see, e.g., Pandey, 1979)–by research focusing on the influence of status signals emitted by direct beneficiaries of compassionate behavior, we caution that the current experiments can only allow firm conclusions about compassionate responding itself (the observed behavioral responses of participants in the field study, assuming that donations are intended to alleviate suffering in the context of poverty and homelessness) and to perceptions of the target (in the perception study) that are elicited by status signaling. Given the precise ways in which participants interacted with the individual (shown) soliciting money in the two studies, we refer to him as the “confederate” in the field study (a term that is inclusive of seeing the individual as a recipient or facilitator of compassionate responding) and as the “target” in our follow-up study (because all perceptions measured in this study were with respect to him specifically). We also consider the issue of whether participants saw themselves as donating to the confederate himself or not, and the implications of this distinction, in the General Discussion.

The field study methodology we employ in our primary experiment represents a key strength in relation to recent research on the topic because it examines compassionate responses, indexed by donation behavior in a real-world giving context, rather than measuring intentions to donate to hypothetical targets or actual donations in computer-mediated interactions. In comparison to much of the prior research investigating similar topics, this methodology also indexes an unambiguous sacrifice on the part of those who respond compassionately (donating one’s own money, cf., Bickman, 1971) in a way that allows for more precise estimation of the degree of differences in generosity (cf., Solomon and Herman, 1977; Goodman and Gareis, 1993), at least in the aggregate. Finally, the context of this field experiment more closely mirrors most of the actual contexts in which individuals have opportunities to enact compassion on a day-to-day basis (but see, Tracy et al., 2018 on the rising relevance of online giving behavior).

## Study 1: A field study of social class signals and compassionate responding

We first tested our central hypothesis in a field experiment that sampled pedestrians on public streets of two major cities. Specifically, we expected passersby in six busy locations in downtown urban areas of the United States to donate more money to a panhandler signaling relatively high status, compared to relatively low status, through clothing (a highly salient method of status signaling employed in previous research; e.g., Bickman, 1971; Kraus and Mendes, 2014).

### Materials and method

#### Participants

Participants for this study consisted of pedestrians in New York City, NY and Chicago, IL that happened to pass a confederate during the course of the study. Spotters, research assistants for the study, were present at each location to record the number of pedestrians (inter-rater reliability *r* = 0.99, *p* < 0.001), defined as individuals passing the confederate on the same sidewalk (*N* = 4,536). In total, 1,996 and 2,540 individuals passed the higher status and lower status confederate, respectively. We arrived at this eventual sample size in an effort to collect as much data as possible. We determined, before each trial began, how long each trial would last, based on the availabilities of the confederate and research assistants. We collected data only during those trials and over the course of the entire trial, except in one instance when the trial was cut short: in one of the higher status trials (Location #4, See Supplementary Figure 1) the confederate was asked by security to leave his position early, and the confederate complied without incident. We excluded one participant, and their second donation, from the analysis because they happened to encounter the confederate during both conditions.^1^ Because we had little control over the eventual number of participants included in the study, we did not designate a target sample size; however, a sensitivity analysis (Faul et al., 2007) determined that the resulting sample size was sufficient to detect a small effect, expressed as a difference in independent means according to an independent-samples *t*-test (*d* = 0.10), with 95% power and a false positive rate of 5% (α = 0.05).

#### Procedure

**FIGURE 1:**
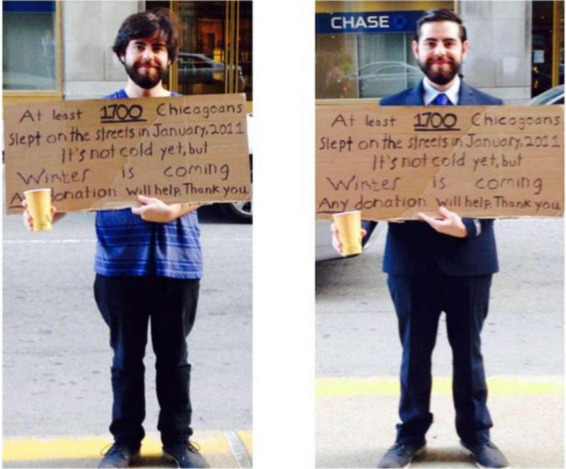
The confederate (the study first author) wearing low (left) and high (right) status symbols at W. Jackson Blvd between S. Michigan and S. Wabash streets. Images are intended for demonstration purposes only (i.e., they do not represent exactly what participants saw). Bennett Callaghan served as stimuli for the study itself.

A single confederate (the study first author) stood at locations where panhandlers and unhoused individuals were previously observed. The confederate wore high or low status clothing–depending on social status condition–and held a cardboard sign with a message about the number of unhoused people in New York or Chicago (depending on location). He used a paper coffee cup for collecting donations and occasionally said “Collecting money to help the homeless” in order to draw attention from pedestrians. Otherwise, the confederate was instructed not to engage with or speak to any passersby (unless they spoke to him first) and to maintain a natural facial expression and tone of voice. The confederate did not display any overt signs of suffering in either condition. The cardboard sign and collection cup were intended to further reinforce a context of low social class and homelessness across conditions (Cuddy et al., 2002). In the lower status condition, the confederate wore jeans and a t-shirt, and in the higher status condition, he wore a business suit, dress shirt, and tie. Additionally, and in order to both amplify the impact of status signaling and make the confederate’s personal appearance more congruent with the relatively higher status signals communicated by a business suit, the confederate in the higher status condition also used pomade to slick back his hair (see Figure 1).

To control the experimental setting as tightly as possible between conditions, data were collected for similar amounts of time, on similar days, and in the same locations in the higher and lower status conditions (see Supplementary Table 1). The locations used in New York City were as follows: (1) East 17th street and Broadway, (2) St. Mark’s Place and Avenue A (near the entrance of Tompkins Square Park); (3) Central Park West between 62nd and 63rd streets; and (4) 56th street and 8th Avenue. In Chicago, they were (5) South LaSalle street between West Lake street and West Wacker Drive and (6) West Jackson Blvd between South Michigan and South Wabash streets. All of these locations were accessible by public transit, and they were sufficiently busy that, in each, very few generalizations can be made regarding those who happened to pass the confederate. For instance, because such locations were public and easily accessible, it cannot be assumed that all passersby tended to share particular sociodemographic characteristics. While we did not record such characteristics, we expect that this procedure sampled a wide spectrum of those one might encounter in a busy section of a major American city. Thus, we expect that these participants represented a diversity of racial, gender, and, importantly, socioeconomic backgrounds. Perhaps with the exception of one (Location #2), however, we do note that each of these locations was situated in or near commercial districts, which may have biased our sample slightly toward those of a somewhat higher SES than average.

In total, the higher status and lower status trials did not differ in terms of the number of participants, *t*(10) = 0.54, *p* = 0.60, duration, *t*(10) = 0.30, *p* = 0.77, start time of the trials (measured in seconds since midnight), *t*(10) = 0.46, *p* = 0.66, the ambient temperature at the start of the trials, *t*(10) = 0.20, *p* = 0.85, or the day of the week on which they fell, χ^2^(3) = 2.67, *p* = 0.45. Finally, there was no correlation between the amount given per participant (the total amount within a trial divided by the number of donors, to account for the fact that later trials contained more participants) and calendar date (with August 5th coded as 0 and subsequent days coded as days since August 5th), *r* (10) = 0.43, *p* = 0.16. Thus, any incidental differences among the trials–other than the status manipulation–are unlikely to account for observed differences. We should note, however, that all trials took place on weekdays, and began between 2:45 PM (at the earliest) and 7:45 PM (at the latest). This is important context to keep in mind, as participants in trials conducted during a typical workday (9:00 AM–5:00 PM) may have been more likely to infer that the confederate himself did not himself have a typical (or perhaps any) job. Supplementary Table 1 in the Supplementary Information provides full trial-level data.

The confederate was assisted at all locations by two trained spotters: research assistants who were tasked with maintaining the safety of the confederate, counting pedestrians crossing on the same side of the street as the confederate (to determine participation in the study), counting the number of people donating, counting the number of people who interacted with the confederate beyond giving money (such as saying something to him, regardless of whether they donated), and handling any interactions with public law enforcement or security. All collected funds were subsequently donated to a local homeless shelter. To comply with Institutional Review Board guidelines, the confederate did not lie to any of the participants by telling them that he was specifically collecting money for himself, but he also did not reveal his status as a researcher and that they were participating in a study. Upon completion of the study, donations in New York were sent to the Bowery Mission^2^ and donations in Chicago were sent to the Chicago Coalition for the Homeless.^3^

Spotters showed high consistency in their coding of relevant variables. Overall pedestrian counts conducted by spotters were identical in all cases, save for one trial (the low status condition for Location #2) where the counts differed only by one. Counts for the number of those who donated (coded as seeing a participant physically give money) were identical for all trials, and the counts for the number of interpersonal interactions differed by one in two trials (the high status condition for Location #5 and the low status condition for Location #6).

Overall generosity in each trial was determined by counting up the total amount donated in United States dollars. In each trial, spotters kept a count of the number of people who donated. When possible (i.e., if participants donated an amount in a large denomination), the research team also kept track of the amount donating in each transaction. When this was not possible, donations where the amount was ambiguous were assigned a constant value calculated based on all the remaining money donated, with these large donations subtracted from the total trial amount, for certain analyses. Interestingly, all of the highest donation amounts of $5 United States (twice, at Location #4) and $10 United States (twice, once at Location #4 and once at Location #6), which could be recorded as discrete donations, occurred in the high status trials.

For the purposes of this study, we define compassionate responding as both the monetary amount and the frequency of donations that the confederate received in each condition. However, we also report on the number of large donations (defined as those $5 and above) and (in the Chicago trials) the number of people who went out of their way to interact with the confederate, whether they donated or not. We kept track of large donations and interaction instances because differences by condition in these two variables could suggest qualitative differences in how participants approached the two confederates. The latter measure also disentangles, to some extent, the degree to which differences in generosity are due to intentions to engage specifically in compassionate responding, rather than general tendencies to approach and interact with the confederate–perhaps due to the potential novelty of a panhandler wearing a suit.

### Results

To test our hypothesis about status symbols and compassionate responding, we first examined the total amount donated to the confederate as a function of social status condition. Given the nature of data collection, we do not have access to individual donation amounts; thus, we first analyze the total distribution of donations across conditions because such an analysis requires the fewest assumptions about the underlying distribution of the data. In total monetary value (i.e., collapsing across trials and without making assumptions about the size of individual donations), the higher status confederate received more than twice as much (2.55 times) money as the lower status confederate over all trials: $54.11 (over the course of a cumulative 3.5 h) compared to $21.15 (over 4 h). A chi-square goodness-of-fit test determined that this distribution differed from that expected by chance, χ^2^(1) = 14.44, *p* < 0.001. The Supplementary Information also provides an additional test, using a general binomial linear mixed model framework, to analyze donations (using an approximate method of apportioning donations) while accounting for the random effect of location. This analysis yielded similar results.

We also examined mean differences as a function of individual donations, using the apportionment strategy described above. While this method imposes additional assumptions, compared to the analysis reported above, about the distribution of the underlying data, it is nonetheless instructive. The mean difference amounted to an average of three cents-per-participant (passerby) across high-status trials (*M* = $0.027, *SD* = $0.37) compared to less than one cent (*M* = $0.008, *SD* = $0.10) across low-status trials. Because these data were unlikely to be normally distributed, we conducted a Wilcoxon ranked-sum (i.e., Mann–Whitney) test with a continuity correction, *W* = 2,521,391, *p* = 0.06, *r* = 0.03 [95% CI: 0.002, 0.06] to compare these means. The Supplementary Information provides an additional test (assuming normality) accounting for the potential moderating influence of city; this analysis did not show any evidence that this effect differed for participants in New York and Chicago.

Follow-up exploratory analyses revealed results that were–though weaker (likely owing to the infrequency with which the focal events occurred)–in line with our hypotheses for the number of donors and the distribution of large donations. For number of donors, we used a 2 × 2 contingency table analysis accounting for the number of participants who did and did not donate within each condition (frequency of donations and percentage of donors, relative to total condition sample, reported in parentheses). Though overall donation rates were low, a Fisher’s exact test (which is suited to dealing with small or unbalanced cells within a contingency table; Fisher, 1922) on this table suggests a greater number of donors in the higher (*N*_*donors*_ = 25; 1.25%), as compared to lower (*N*_*donors*_ = 18; 0.71%), status trials. Directionally consistent with our hypotheses, this analysis revealed that higher status trials had a marginally significant higher proportion of donors than lower status ones, *p* = 0.066. We used the same contingency table analysis to examine the distribution of large donations of $5 and $10, which occurred only four times in total (all in the high-status condition). The degree to which the higher status trials dominated these large donations also differed significantly from what would be expected by chance, *p* = 0.04. However, the same analysis applied to the number of interactions with the target (e.g., saying something to him, regardless of whether they donated) did not differ by condition, *p* > 0.99, suggesting that the observed differences in donations are not solely attributable to people noticing and approaching the higher status confederate more often. Pedestrians interacted with the higher status (*N*_*interactions*_ = 12; 0.60%) and lower status (*N*_*interactions*_ = 15; 0.59%) confederate at roughly equal rates. Thus, we find some evidence that the higher status confederate not only collected a larger total amount than the low status confederate, but we also find some evidence that individuals were more likely to donate to the former than the latter, especially with respect to large single donations of $5 or more.

### Discussion

In Study 1, signaling relatively high, compared to low, status drew both more and greater donations to a panhandler from passersby in major urban areas. This advantage amounted to a more than two-fold increase in overall donations (according to an analysis that imposes the fewest assumptions) and emerged despite equivalence across conditions on important variables such as the length of the trials, the number of participants per trial, and the ambient temperature. Thus, this field study supports our hypothesis that symbols of higher social class (expressed through sartorial displays) influence compassionate responding. Notably, preliminary evidence suggests that these signals did not operate simply by way of increased noticeability or approachability, as indicated by the lack of a difference in tendencies to approach and interact with the panhandler.

The strongest result obtained in this study is that the confederate collected more money, in aggregate, while signaling relatively higher SES. As noted, this investigation of aggregate effects–assessed by analyzing the degree to which the total distribution of donations across the two conditions differed from one expected by chance–was simultaneously well-powered to detect such an effect and imposed the fewest assumptions about the underlying structure of the data (which was, due to the nature of data collection, unobservable). However, some of the specific analyses (i.e., an analysis approximating individual donations assuming fixed variance within trials and the analysis for total number of donors) fell short of conventional cutoffs for statistical significance (Cohen, 1994).

One explanation for why these analyses fell short of these cutoffs is that they lacked statistical power due to the (in)frequency of the focal events themselves: for instance, fewer than 2% of the roughly 2,000 participants in each condition (fewer than 20 donors per condition) actually donated to the confederate. Thus, even though we collected a large sample, instances of compassionate behavior that are operationalized by counting discrete events, such as donation, may require an even larger sample to detect robust differences using the current methodology. Future researchers, then, may attempt to collect larger samples in a similar experiment, either by conducting more trials or by lengthening the trials themselves. Alternatively, future researchers might employ more salient methods of attracting attention from passersby (provided they remain constant across conditions) in order to increase engagement from passersby and, hopefully, increase overall donation rates. However, the mere rarity of these events likely does not fully explain why some analyses produced stronger effects than others. For instance, only four individuals, in total, donated amounts of $5 or larger. However, the distribution of these donations was so extreme, that analyses on these donations nonetheless produced significant results. Unquestionably, these large donations represent outliers, which likely further shifted the distribution of donations away from normality and informed the decision to adopt a non-parametric, rank-based test for comparing donations at the individual level. Such a test is robust to outliers, as compared to a more standard parametric one (e.g., one assuming a t distribution; Zimmerman, 1994), but it also necessarily lessens the distance between common donation amounts and larger donations, which also likely contributed to the lack of statistically significant results in this analysis.

Other methods of modeling these data (such as the negative binomial linear mixed regression reported in the Supplementary Information, which also accounts for the random effects of trial location) may lead to more statistically robust results, but they also require unverifiable assumptions about the underlying data. Thus, the conclusions one might draw from these results likely depends on how one treats these large donations and apportions the remaining donation amounts among remaining donors. It is also likely that the significant differences we observe are in large part driven by these large donations. As evidenced by the fact that all of these donations occurred in the higher status trials, we see these donations as carrying meaningful information about the compassionate responding of participants (rather than reflecting mere statistical noise). However, as discussed more in depth in the General Discussion, their presence also raises interesting questions about how and for whom status signaling might impact compassionate responding. For instance, it is possible that our manipulation of status more precisely influences extreme instantiations of compassionate responding or that its influence is confined to particular individuals (e.g., those who are predisposed toward more extreme compassionate responding in the first place, or wealthier individuals for whom a larger donation represents less of a sacrifice).

Study 2 was designed to investigate the potential mechanisms by which high status signaling may have elicited greater generosity. In particular, it tests the plausibility of the theoretical account posed at the outset: that relatively low status signals create a pattern of social perceptions that dampen compassionate responding.

## Study 2: Appraisals for targets of compassionate responding

An online follow-up experiment examined the perceptions associated with targets based on the confederate employed in the field experiment. Study 2 tested whether those signaling relatively low status were also seen as less competent, less warm, less similar to the self, and less human–all qualities that decrease compassionate responding and would comport with our proposed social perception account for the field study results. In addition, this follow-up study further assessed an alternative, novelty-based explanation for these results: that a target asking for money while signaling relatively high status simply attracted more attention than his counterpart.

### Materials and method

#### Participants

We recruited 504 online participants from Amazon’s Mechanical Turk (51% self-identified as male, one participant did not self-identify). Roughly 75% of participants identified primarily as White/European-American, 8% as African-American, 9% as Asian-American, and 6% as Latino/a. We collected at least 100 participants per condition in order to detect an effect size of *d* = 0.40, the average effect size in social psychology (Richard et al., 2003) with 80% power. We attempted to exceed this benchmark, while remaining within financial constraints. We intentionally recruited a larger sample size than that which is required to detect an effect of *d* = 0.40 in order to detect smaller effects, should they arise, to provide more precise point estimates of any effect size, to account for potential attrition, and because we measured multiple dependent variables–which can inflate the family wise error rate.

Three participants did not complete an attention check, and 15 were excluded after failing an attention check. Specifically, we showed participants pictures of the confederate from Study 1 (see Supplementary Figure 1A and Procedure for more details) and asked them to indicate what the target wore out of the following four options: (A) “Business suit,” (B) “T-shirt and jeans,” (C) a “Hawaiian shirt,” and (D) none of the above. As expected, the majority of those in the relatively low and high status conditions, respectively, chose options (B) and (A). Because they were obviously incorrect, we excluded one participant in the lower status condition who chose option (A), two participants in the higher status condition who chose option (B), and six participants who chose option (C). However, we perhaps overestimated the consistency with which people would describe the sartorial choices of the target, as 29 and four people in the lower and higher status conditions, respectively, chose option (D). We did not exclude participants who chose this option because doing so would introduce differential attrition and because participants may have subjectively considered the target’s clothing to be something other than a t-shirt and jeans or a business suit while still recognizing that the two wardrobes signaled differential status (as was later confirmed by a manipulation check). Nonetheless, the results remain largely similar with participants who chose “none of the above” excluded (see Supplementary Information). Thus, we analyzed responses from 492 participants in total and did not exclude any other participants, except in cases of missing data.

#### Procedure

All participants completed a survey that ostensibly aimed to investigate “perception” and that involved “looking at images… and giving us your feedback.” After providing informed consent, participants viewed images of the confederate from Study 1 and responded to a set of questions concerning him. The target signaled higher or lower status by appearing dressed in a business suit or jeans and a t-shirt, as in Study 1 (see Supplementary Figure 1A). Participants in this study were first briefly (3 s) exposed to a wide-shot photograph of a street in Champaign, IL that depicted the confederate panhandling on a populated street while signaling higher or lower status. The images were manipulated such that everything except the clothing of the target was identical across conditions (see Supplementary Figure 1B). Participants were subsequently asked to list up to five things they saw in the photograph.

After listing these items, participants saw a second, larger photograph of the target in high or low status clothing (see Supplementary Figure 1A). Participants then made social perception judgments regarding stereotype content and person perception based on this latter image, which appeared and remained at the top of each page to assist in making judgments. In randomized order, participants were asked to judge the target, absent all other information apart from his physical appearance, on a number of social attributes, including his perceived competence, warmth, interpersonal closeness, and humanity. These constructs were chosen due to their relevance to both status and compassionate responding on the part of others (descriptive statistics, overall and by condition, for each of these variables is available in the Supplementary Information; see Supplementary Table 2). The design of this study was fully between-subjects and the status condition of the target was consistent across the brief exposure and perception tasks, meaning that participants only saw the high status or low status target throughout.

To determine the success of our social status manipulation, participants also ranked the target they saw on a ten-point scale of subjective SES used in prior research (Adler et al., 1994; Kraus et al., 2013) wherein participants ranked the target on a 10-rung ladder representing ascending levels of education, income, and occupation status in the United States. Based on this measure of social status position in society–and consistent with our expectations–the higher status target (*M* = 3.53, *SD* = 1.89) was judged as considerably higher in social status than the lower status target (*M* = 2.44, *SD* = 1.65), *t*(482.34) = 6.82, *p* < 0.001, *d* = 0.61 [95% CI: 0.43, 0.80]. However, and consistent with the experimental context of poverty, both the relatively high and low status targets were judged to be low in status relative to the scale midpoint *t*(246) = −12.20 and *t*(244) = −24.24, respectively, both *p*s < 0.001.

#### Materials

##### Noticing the target

We designed the brief exposure task as a way to determine whether the higher status target was more novel or attracted more attention than the lower status one (perhaps due to expectation violations of a denigrated group member being dressed in higher status clothing). The first author used the responses to the brief exposure task to determine whether or not each participant noticed the target (the coder was blind to condition except in cases where their answer referred to what the confederate was wearing, in which case the fact of the participant noticing the confederate is unambiguous). To do so, the coder read the (up to five) things that participants listed having seen, and judged whether or not they referred to the target; if a participant acknowledged the target at least once over the course of their responses, that participant was given a score of 1 (and a score of 0 otherwise). For example, responses such as “tree” or “man with backpack” would not substantiate noticing the target, whereas responses such as “man asking for money” or “panhandler” would. The third author (also blind to condition) independently coded a random subset of 99 responses and scored them in the same manner. The two coders showed adequate (Landis and Koch, 1977) reliability (κ = 0.61).

##### Warmth and competence

In making their social perception ratings, participants were asked to indicate how much a number of words described the target on 0 (Not at all) to 100 (Totally) slider scales. We expected participants to see the lower status target as less warm (i.e., “friendly,” “trustworthy,” “good-natured,” “well-intentioned,” “warm,” and “sincere”) and competent (i.e., “competent,” “intelligent,” “capable,” “confident,” “efficient,” and “skillful”) than the higher status target, according to measures drawn from previous research (Cuddy et al., 2002, 2008). Both of these scales displayed strong reliability (α = 0.95 and 0.94, respectively).

##### Similarity to the self

Given our prediction that participants would tend to “other” the target–especially the lower status one–and distance him from the self, we measured self-other similarity using the Inclusion of Other in Self (IOS) scale (Aron et al., 1992). Participants indicated which pair of seven increasingly overlapping circles labeled “Self” and “Other” most closely resembled their “relationship with people like the person pictured above”; higher scores indicate greater self-other similarity.

##### Ascribed humanity

We hypothesized that participants would also tend to see the lower status target as less human than the high status target–that is, lacking traits typically associated with humanity and personhood and having traits associated with animality (Laughnan et al., 2014). Consistent with previous research, we refer to this construct as “ascribed humanity” (Martinez et al., 2011). An ascribed humanity index consisted of (a) a shortened version of a humanity scale, asking participants to indicate how much they thought a number of words (e.g., “person,” “citizen,”) described the confederate, (b) a reverse-coded shortened animality scale (e.g., “wild,” “untamed”) and (c) their agreement with how much the target embodied personality traits typically considered to be uniquely human: openness to experience (e.g., “open to new experiences, complex”) and conscientiousness (e.g., “dependable, self-disciplined”). These items were drawn from previous research and averaged into a single scale, also consistent with this previous research (Martinez et al., 2011). These items were measured on the same 0–100 sliding scale used to assess perceived warmth and competence, and the scale displayed strong reliability (α = 0.84).

### Results

#### Brief exposure task

Participants were more likely to notice the target in the lower status (87.35%) than in the higher status (74.49%) condition, χ^2^(1) = 13.15, *p* < 0.001, as determined by the coding of their responses and a chi-square test of association. These results echo those from Study 1 in that the higher status target was apparently not more noticeable than the lower status target. If anything, the former was less noticeable than his counterpart.

#### Social perceptions

**FIGURE 2:**
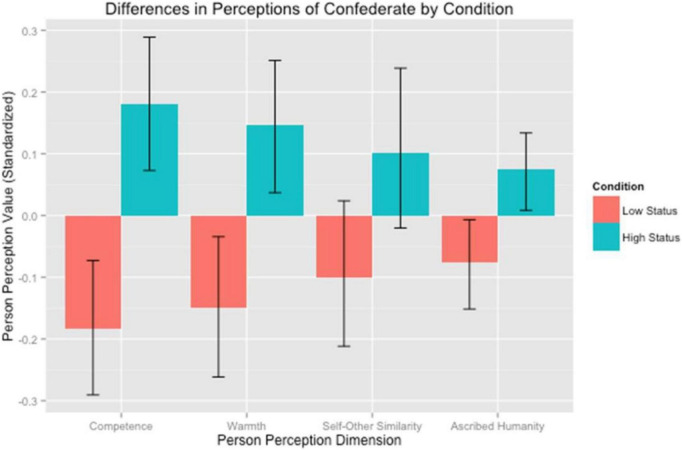
Standardized mean perceptions of higher and lower status targets on dimensions of competence, warmth, self-other similarity, and ascribed humanity. Error bars represent bias-corrected and accelerated (BCa) bootstrapped 95% confidence intervals with 5,000 replications. Bennett Callaghan served as stimuli for the study itself.

A Multivariate Analysis of Variance (MANOVA) revealed that the social status manipulation influenced the hypothesized perceptions of the target in the expected manner, Wilk’s λ = 0.95, *F*(4,486) = 5.96, *p* < 0.001. Specifically, participants judged the higher status target as more competent, *F*(1,489) = 21.35, *p* < 0.001, *d* = 0.42 [95% CI: 0.24, 0.60], warmer, *F*(1,489) = 13.42, *p* < 0.001, *d* = 0.33 [95% CI: 0.15, 0.51], more similar to the self *F*(1,489) = 5.05, *p* = 0.025, *d* = 0.20 [95% CI: 0.02, 0.38], and more human, *F*(1,489) = 9.20, *p* = 0.003, *d* = 0.27 [95% CI: 0.10, 0.45] than the lower status one (see Figure 2; Supplementary Table 2).

As might be expected given our theoretical background, each of these perceptual variables also correlated positively with subjective SES, which was the manipulation check and the measure of perceived status for the target (see Supplementary Table 3).

### Discussion

Study 2 builds upon the results of Study 1 by outlining the patterns of social perception that guide preferences to share resources with individuals signaling higher social class in interpersonal contexts. Specifically, participants judged the relatively low status target to be less warm, less competent, less human, and less similar to the self than the relatively high status one. Consistent with initial expectations, however, both targets were seen as generally low in status. The results of Study 2 also further reduce the likelihood of a mundane explanation for the tendencies observed in our field experiment: that the higher status confederate drew more compassionate responding simply by appearing more novel and drawing more attention. That participants were more likely to indicate noticing the lower status target runs counter to such an explanation and perhaps suggests greater vigilance of low status targets, who are often seen as potentially threatening (Major and O’Brien, 2005).

Instead, the social perceptions engendered by the lower status target were largely consistent with research showing that people perceive extremely low status groups in society, relative to their high status counterparts, as less warm, less competent (Cuddy et al., 2002, 2008), less similar to the self (Bastian and Haslam, 2010; Kraus and Keltner, 2013), and less human (Laughnan et al., 2014). Thus, these results provide evidence for the multiple psychological perceptions that arise from status symbols and commonly precede expressions of compassion.

## General discussion

As economic inequality rises in many parts of the world, and countries such as the United States roll back social safety net programs (Piketty, 2014), the responsibility for dealing with inequality’s deleterious impacts (Wilkinson and Pickett, 2009) has increasingly fallen to economically precarious individuals themselves or to private citizens exercising compassion, defined as concern for the suffering of others and the motivation to help improve their circumstances (e.g., Goetz et al., 2010; Gilbert, 2017; Mascaro et al., 2020). Building on prior research and theorizing in the rich tradition of research on sympathy, empathy, and compassion (Batson et al., 1989; Cialdini et al., 1997; Oveis et al., 2010), the current research examined the tendency for people to respond compassionately (or not) in the presence of those who were apparently suffering or, at least, made salient a concern with suffering related to poverty and homelessness (i.e., a panhandler), in two cities in the United States. The current research suggests that people respond more compassionately, and perceive such individuals more favorably, when they signal higher–relative to lower–social status through physical appearance. This pattern of results arose even though all confederates and targets appeared to be generally low in status, and it arose in an experimental, but ecologically valid, context where participants shared their own money.

This research also contributes to a longstanding body of research suggesting that non-verbal status cues influence behavior on the part of others (e.g., Bickman, 1971; Tracy et al., 2018). That symbols of high social class more than doubled the donations of pedestrians over a 4-h period indicates their power in shaping initial judgments of others’ basic human traits and in eliciting compassionate responses in everyday life. Importantly, our results align with past theory and research suggesting that high status signaling provides many direct benefits to individuals, including grooming and mating partners in non-human primates (Sapolsky, 2004). This research adds received generosity, among humans, to this list of benefits.

Interestingly, mere novelty and noticeability of the higher status confederate do not seem to explain observed differences in generosity. In the field experiment, the mere frequency of the interactions did not differ by condition; in Study 2, in fact, participants were in fact more likely to attend to the lower status target. Instead, the quality of these interactions and their outcomes (as indicated by the analysis of extreme donations) differed. Anecdotally, this qualitative distinction bears out. When people did go out of their way to speak to the confederate, the higher status one received comments such as “I usually don’t give money to people on the street, but you seem like a nice guy.” In one case, a pedestrian (also donned in a business suit) even dropped a business card into the higher status confederate’s collection cup–a tacit invitation for the confederate to seek employment, rather than a trivial one-time donation.

As discussed, the large donations of $5 or $10, given their size and exclusive presence in the relatively higher status trials, likely contribute substantially to some of the effects we observe in the field study. Much like the interactions sketched above, these donations might also represent a qualitative shift in how donors approached the situation: they may have donated $5 or $10 in the hopes of more effectively meeting the confederate’s immediate perceived needs, as such an amount would be more appropriate than more common donation amounts (e.g., $1 or less) for most self-care and survival needs, such as purchasing a meal. Thus, these donations might be particularly representative of compassionate responding insofar as they are intended to effectively and (depending on participants’ construal of the situation) immediately alleviate suffering. However, they also suggest the possibility of theoretical accounts we did not fully theorize. For instance, it is possible that status signaling is most effective at eliciting high-variance responding; in other words, signaling higher status might not strongly impact tendencies to engage in compassion in general, but, rather, impacts tendencies to engage in extreme–as defined in relation to more typical donation amounts–acts of compassion (again, however, use of the word “extreme” might be misleading, as these donations might also be described as simply independently sufficient to meeting the goals at hand).

It is also possible that this pattern of results reflects an unobservable moderation effect. Perhaps, for instance, the effects of status signaling are most pronounced among those who are more inclined to acts of extreme generosity to begin with. Alternatively, this effect might be attributable to the presence of stronger effects among participants who are higher in SES themselves. The design of the field experiment study did not allow us to assess the SES of passersby, and, thus, whether participants’ own social class characteristics contributed to decisions to respond compassionately to the confederate. As indicated by the overall low levels of subjective SES attributed to the target in the perceptual study, it is likely that the higher status confederate was perceived as closer to participants, in terms of socioeconomic standing, than the lower status confederate across the board (excluding those who are themselves poor or unhoused). Still, however, the perceptual study does suggest meaningful differences in self-other similarity according to status signaling condition, and the possibility that signalers who better “match” the status of perceivers benefit from even greater compassion than those who merely signal higher status has received mixed empirical support (see, e.g., Goodman and Gareis, 1993). Thus, it is possible that high status signals appealed specifically to passersby of particularly high SES and who, due to greater access to financial resources, may have stood to lose less through larger donations or simply regarded higher amounts of money as an appropriate default for donation (as a proportion of the money they had on hand, for instance). Though it may be difficult to measure individual differences such as predispositions toward extreme generosity in a field study context, future replications of this research might employ methods of subjectively coding participant SES (e.g., Bickman, 1971) or systematically varying the SES characteristics of the research sites (e.g., Goodman and Gareis, 1993) in order to determine the regularity with which these extreme donations occur and whether they are given disproportionately by those of higher socioeconomic standing.

Together, these qualitative experiences and extreme donation profile provide some support for the general pattern observed in Study 2, and support a central tenet of theories of compassion: that compassionate responding hinges on the reputation of targets, especially with respect to their likelihood of engaging in reciprocal cooperation with other prosocial individuals (Goetz et al., 2010). The present research adds signals of social class as a possible cue that reliably elicits such reputational perceptions.

Moreover, high status signals increased specific judgments of competence, trustworthiness, humanity, and self-other similarity. Thus, the results of the current studies suggest that poor individuals who adopt these symbols might be seen as more effective at converting gifts into intended outcomes (such as personal advancement or care), as less likely to engage in behaviors that might be seen as making them blameworthy for their plight (e.g., drug or alcohol use; see Goetz et al., 2010), and as more likely to use those gifts for intended means rather than as a strategy to accrue undeserved wealth. In short, such signals may make one appear more deserving of compassion (Goetz et al., 2010).

A closely related alternative explanation for the current set of results, which more strongly emphasizes the perceived ability (rather than the inclination) to engage in future prosocial behavior by the confederate, is that participants were more likely to see the higher status confederate’s need state as temporary, rather than chronic. Consistent with certain evolutionary accounts of reciprocal altruism (e.g., Sugiyama and Sugiyama, 2003; Tracy et al., 2018), the perceived combination of high temporary need and high baseline competence may have biased individuals toward helping the higher status confederate in his time of need because he was perceived as more able to help others, or “pay it forward,” when he had the opportunity to do so. Given that the high and low status targets were strongly discriminated along the lines of competence, this alternative explanation is plausible. Future research is needed, however, to determine whether such perceptions of ability to engage in future acts influence compassionate responding independent of perceptions of deservingness.

In a similar vein, our field study operationalizes compassion as costly helping behavior–a common method of doing so within the social-psychological literature and one that avoids many of the biases inherent in self-report measures (Mascaro et al., 2020). Our second study also includes a number of social perceptions that index deservingness, an antecedent to compassion in prevailing theoretical accounts of the construct (e.g., Goetz et al., 2010). While this research demonstrates the influence of status signaling on theoretically important perceptions of a target (Study 2) and responses toward a confederate (Study 1), this research does not measure compassion, as a subjective psychological state, directly. Nor does the second study measure compassionate responding directly, as in Study 1. Thus, the two studies taken together show a pattern that is consistent with a theoretical account emphasizing compassion: one in which status signaling affects particular theoretical antecedents of compassionate responding (i.e., warmth, competence, self-other similarity, and ascribed humanity), which then influence compassion and compassionate responding. However, these results do not necessarily confirm that status signaling directly influences perceptions linked to deservingness and, subsequently, compassion and compassionate responding.

To address this theoretical gap, future research might attempt to measure compassion directly and demonstrate that signaling relatively higher (as compared to lower) status–by way of heightened perceptions of deservingness–heightens self-reported compassion for those suffering in the relevant context as well as subsequent compassionate responding (i.e., donations). In doing so, researchers should be mindful of best-practices in the measurement and definition of this complex emotion (Gilbert, 2017; Mascaro et al., 2020). For instance, such research might attempt a multi-method approach to conceptualizing and measuring compassion that synthesizes quantitative reports of one’s own and others’ mental states, physiological measurements, and observations of behavior (e.g., Mascaro et al., 2020). Additionally, such research might take care to distinguish compassion from subjective and emotional states–such as distress, sadness, and love–that are sometimes used interchangeably with compassion in the literature (e.g., Goetz et al., 2010; Gilbert, 2017). Second, in order to test the full theoretical model we have proposed here, future research should manipulate status signaling and measure both the antecedents we propose and compassion (or compassionate responding) within the same study. Such a study could at least determine whether the key variables related to deservingness mediate the effect of status signaling on compassion. Ideally, future research could also manipulate these mediators to establish a truly causal chain of effects (Spencer et al., 2005).

It is also possible, however, that conditional differences in confederate behavior contributed to differences in generosity on the part of passersby. The confederate was not blind to condition or hypotheses, and previous research suggests that donning high status sartorial signals can change the behavior of even naïve participants (Kraus and Mendes, 2014). Though this is a possibility, we minimized this likelihood by having the confederate behave consistent with standardized instructions. Moreover, that a follow-up study elicited theoretically relevant patterns of perception from passive observers suggests that the effect of status signaling on generosity observed in the field is at least partially driven by perceiver judgments. Finally, even if the behavior of the confederate did subtly differ between conditions, such subtle differences would need to compete with the cacophony of stimuli that individuals normally encounter when walking down a busy street in New York or Chicago, so the context in which we chose to conduct our field experiment also mitigates concerns with experimenter effects.

Indeed, it was partially because we expected multiple competing demands on the attention of passersby that we chose to manipulate comparatively obvious visual cues (combined with spoken statements to draw attention), rather than other cues that also signal status, such as vocal pitch (e.g., Gregory and Webster, 1996), accent (e.g., Labov, 2006; Kraus et al., 2019), or cultural signifiers of aesthetic taste (Bourdieu, 1984). Nonetheless, these other modalities represent interesting potential avenues for future research.

Similarly, those who did attend to visual cues of status also likely perceived other superficial but potentially important characteristics, such as those that indicate membership in particular social identity groups. It is interesting to speculate about how these other characteristics of the confederate (i.e., an individual generally perceived to be White and male) may have impacted the effect of status signaling on compassionate responding. For example, membership in other social categories might modify the results observed in these experiments. Theoretical accounts suggest that symbols of social status influence perception similarly across race and gender (Major and O’Brien, 2005), but previous research also finds that social status and race or gender may interact in subtle ways to produce marked differences in status-linked outcomes, such as health and mortality rates (Case and Deaton, 2015) or experienced bias and discrimination (e.g., Goff and Kahn, 2013; Rivera and Tilcsik, 2016). Future research would need to determine if high status symbols confer the same benefits to members of other intersecting social groups as they apparently do for White men.

Future research might also measure how different characteristics of the giving context moderate how status symbols influence outcomes. For instance, some research has found that in contexts where individuals are already motivated to engage in prosocial behavior and are deciding how to distribute their resources, symbols of high status are negatively related to the receipt of altruism (Tracy et al., 2018). These researchers suggest that opposite patterns of effect with respect to status and altruistic behavior might arise depending on whether potential actors are deciding to engage in altruistic behavior in the first place or are deciding how to engage in such behavior. We echo these researchers’ calls for further investigation into this distinction as a potential moderator of the effect of status signaling on compassionate responding (p. 527). We also note that our results regarding the influence of status signaling manipulations on compassionate responses are perhaps bounded to compassionate responding in contexts involving the alleviation of suffering related to poverty and homelessness and to such responses enacted through brief, interpersonal exchanges. Thus, we caution generalizing these results to compassion directed toward other ends or within impersonal contexts, such as online behavior (see, e.g., Tracy et al., 2018).

Finally, we also acknowledge some ambiguity with respect to how participants themselves interpreted donating in the field experiment. As noted, the confederate only told participants that collected funds would be donated to charity if they had asked; few people interacted directly with the confederate in this way, and this pattern did not differ by condition. However, because we were constrained by ethical considerations in terms of what we could tell participants and the field context of the experiment made us unable to probe participants about their inferences regarding the confederate at the time they decided to donate (or not), we still do not know (as discussed) whether individual participants perceived the confederate as the primary benefactor of their donations or as an intermediary.

Even for participants operating under the latter assumption, however, the relevant behavior of donating nonetheless reflects the broader construct of compassionate responding, as those who donated were either donating directly to the target or helping him in his objective to raise money for charity (a goal that is aligned with the reduction of suffering). To this point, previous research has treated explicit contributions to third-party charities as an index for helping behavior directed toward a confederate (Pandey, 1979), and even those who donated under the assumption that the funds would be donated placed significantly more trust in the higher status than the lower status confederate–despite the lack of any guarantee the money would go to charity. Again, such a result is consistent with our overall theoretical expectation that relevant compassionate responding would be directed toward those presumed to be more honest and prosocial themselves, and it would at least appear that signaling status influenced decisions to engage in costly helping behavior (likely driven by differential patterns of social perception) regardless of how participants interpreted the situation. Still, the influence of status signaling on compassionate responding might depend on whether those signaling higher status themselves or third parties are the primary beneficiaries. Future research might investigate this distinction more explicitly.

These limitations and open questions notwithstanding, this research adds to existing models that highlight compassion, sympathy, and perceptions of deservingness as primary causes of compassionate responding (e.g., Goetz et al., 2010). Importantly, our results suggest that social status–and its accompanying interpersonal judgments–enters prominently into such processes. Ironically, low status individuals who appear to need the most help may end up receiving less of it than those who appear higher in status and more abundant in resources.

These results also have direct implications for rising levels of economic inequality in society. Given research suggesting that economic inequality and its negative consequences increase when social status is more visible (Nishi et al., 2015; DeCelles and Norton, 2016), the current findings suggest that status symbols expressed through sartorial displays or other non-verbal behaviors are potential mechanisms for the perpetuation of economic inequality. We found that even among those engaging in ostensibly selfless behavior, individuals were more likely to enter into economic relationships with others who appeared higher, rather than lower, in social status. Given the high degree to which neighborhoods, professional networks, and daily life are stratified by social class, behaviors guided by status signaling can accrue and concentrate wealth and opportunity among a privileged few–further perpetuating inequality (see also Kraus et al., 2019).

These results may also hold implications for addressing economic inequality on a broader societal scale. As indicated by similar research in this domain, cross-status interactions in everyday life can perpetuate inequality by impacting support for social policy aimed at addressing it (Sands, 2017). Nonetheless, such policies are arguably likely to garner the most efficient redistributive outcomes, especially when one considers the alternatives. If subtle interpersonal cues, like clothing or similar indicators of status, shape the behavior of individual actors outside the context investigated in the current research, mechanisms of redistribution that rely on idiosyncratic preferences or the behavior of well-meaning individuals more broadly–such as large donations from wealthy donors to particular individuals or organizations–may be inefficient or underserve those who need the most assistance, whether such needs are met directly or through intermediaries (e.g., charities).

Those from denigrated groups, such as those suffering from homelessness, need monetary assistance despite lacking the ability to transmit status symbols that, as our results suggest, may make certain forms of compassionate responding (i.e., spur-of-the-moment donations) more likely. Moreover, not all charitable organizations aimed at helping such individuals may be equally adept at appealing to wealthy donors or motivating such individuals to donate in the first place. Depending on how far one may extrapolate the results reported here, our research suggests that such a process might require an understanding of how to leverage high status signals (on the part of charities themselves) or how to portray those in need in ways that emphasize their humanity, warmth, competence, and similarity to potential givers. By contrast, codified inequality-reducing policies (such as progressive taxation) do not rely on the generosity of individuals to meet their aims. Unfortunately, even well-meaning generosity, if dispatched at the level of individuals, may be biased by processes of person-perception that direct resources on the basis of attributes other than who is most needy or how resources can best be distributed.

## Conclusion

We found that individuals adopting symbols of higher social class were viewed more favorably by and elicited more compassionate responding (i.e., prosocial behavior) from strangers than those adopting lower social class symbols. These findings suggest the power of status symbols to shape our impressions of others–including the poor and needy–and they highlight how rapidly these perceptions have the potential to shape our social judgments and tendencies to meet suffering with compassionate responding. Understanding the role of status symbols in shaping initial judgments of others has direct implications for bridging divides between the rich and poor in society and, potentially, for shifting broader political attitudes about the causes and consequences of wealth and poverty.

## Data availability statement

The datasets presented in this study can be found in online repositories. The names of the repository/repositories and accession number(s) can be found below: https://osf.io/bxw7g/?view_only=dd6aa7803dce4debb5219bc2b5dd2244 (Open Science Framework).

## Ethics statement

The studies involving human participants were reviewed and approved by University of Illinois, Urbana-Champaign Institutional Review Board. Written informed consent from the participants’ legal guardian/next of kin was not required to participate in this study in accordance with the national legislation and the institutional requirements. Written informed consent was obtained from the individual(s) for the publication of any potentially identifiable images or data included in this article.

## Author contributions

BC and MK designed the experiments, collected the data, analyzed the data, and contributed to the manuscript. QD aided interpretation of results and contributed to the manuscript. All authors contributed to the article and approved the submitted version.

## Footnotes

1 That this individual was exposed to both conditions only became clear when they approached the confederate during the higher status trial (after encountering him during the lower status trial) and asked why he was dressed differently and what he was doing. The confederate then told this participant the truth regarding the study. We retained this participant for the purposes of the low status trial, both because it was not possible to determine which participant they were and how much (if any) money they donated and because they had not yet become aware that they were taking part in a psychological study. No other individuals approached the confederate in this manner, so we cannot be certain that this participant was the only one who was exposed to both conditions; However, given the number of participants within each trial and the fact that no two trials occurred in the same location on the same day, it would seem unlikely that the number of individuals who encountered the confederate twice–much less those who happened to encounter the confederate once in each condition–would be numerous enough to appreciably influence the results.

2
https://www.bowery.org/

3
https://www.chicagohomeless.org/
