# Supplementary material for: The influence of signs of social class on compassionate responses to people in need
Source: Front Psychol. 2022 Aug 25;13:936170. doi: 10.3389/fpsyg.2022.936170 (PMC9455153; doi:10.3389/fpsyg.2022.936170)
Supplement: Supplementary file 1 [file Data_Sheet_1.pdf]

The Influence of Signs of Social Class on Compassionate Responses to People in Need

Bennett Callaghan[1]*, Quinton M. Delgadillo[2], & Michael W. Kraus[3,4]

Affiliations:
[1] The Graduate Center, City University of New York, New York, NY U.S.A
Email: Bcallaghan@gc.cuny.edu
ORCID: https://orcid.org/0000-0001-6227-7708

[2] Columbia University, Business School, New York, NY U.S.A
Email: QDelgadillo24@gsb.columbia.edu

[3] Yale University, School of Management, New Haven, CT U.S.A
Email: Michael.kraus@yale.edu
ORCID: https://orcid.org/0000-0001-6320-4965

[4] Yale University, Department of Psychology, New Haven, CT U.S.A

*Correspondence to:

City University of New York, Graduate Center
Stone Center on Socio-Economic Inequality
365 5th Ave.
New York, NY 10016
Phone: (347) 834-7696
Email: Bcallaghan@gc.cuny.edu

Supplementary Information

A

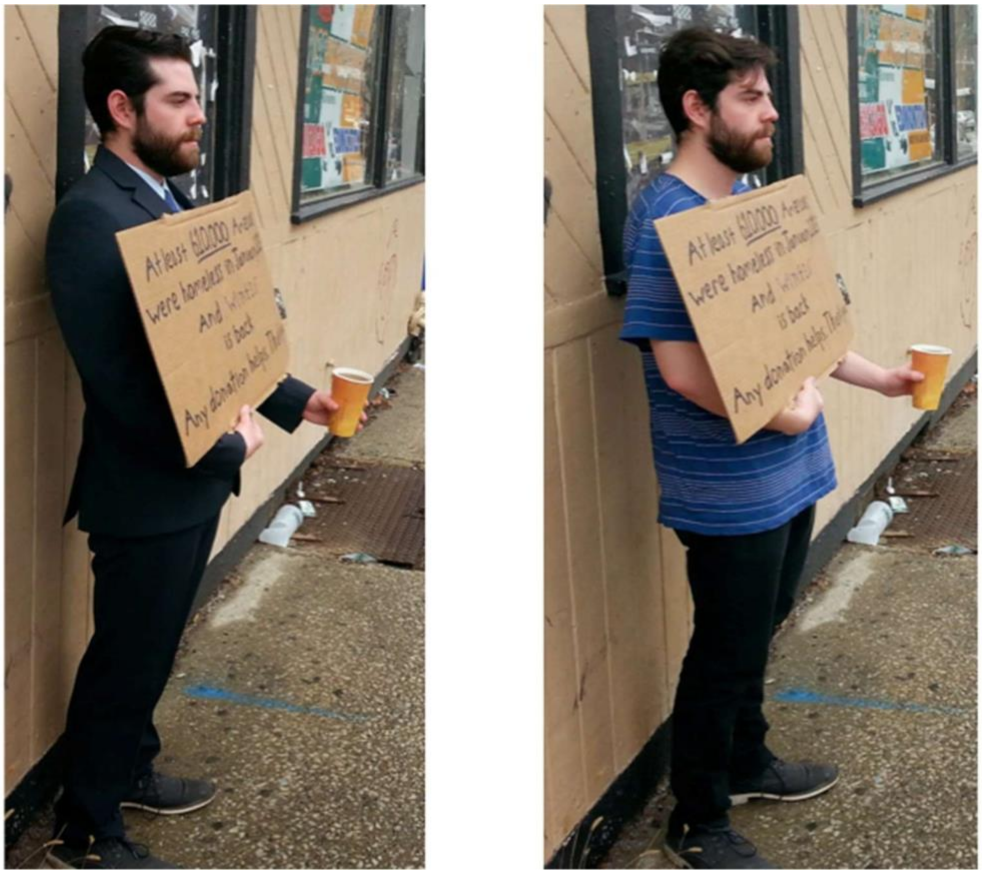

B

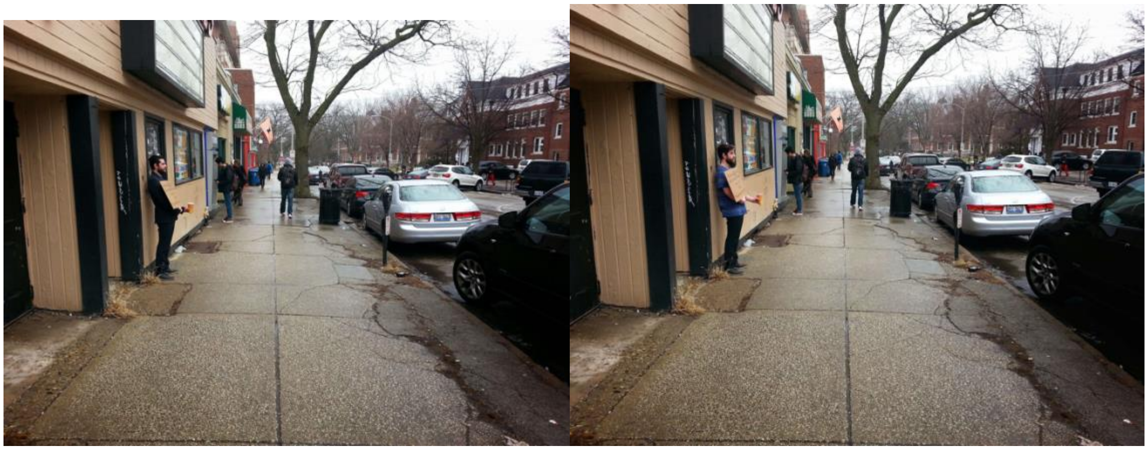

Figure S1. Pictures of the high and low status target used in Study 2 for (A) trait ratings and (B) the brief exposure task attention task.

Table S1

Trial Data and Characteristics (Study 1)

City	Location	Status Condition	N[a]	Number of Donors	Amount Collected (Dollars)	Start Time	Duration[b]	Date	Day	Temperature (°F)[c]
New York	1	Low	259	1	1.00	18:45	15	8/5/14	Tuesday	79
		High	184	1	0.87	17:10	15	8/6/14	Wednesday	82.9
	2	Low	113	3	2.65	19:45	15	8/5/14	Tuesday	78
		High	110	2	2.00	18:05	15	8/6/14	Wednesday	82
	3	Low	228	2	1.50	18:00	15	8/7/14	Thursday	80.1
		High	231	4	3.16	18:00	15	8/14/14	Thursday	75.9
Chicago	4	Low	273	6	5.50	18:30	45	8/7/14	Thursday	79
		High	165	4	20.40[d]	18:40	25	8/14/14	Thursday	75.9
	5	Low	900	4	7.00	16:35	75	9/17/14	Wednesday	70
		High	635	5	8.17	16:40	65	9/19/14	Friday	75.9
	6	Low	766	2	3.50	14:45	75	9/26/14	Friday	79
		High	672	9	19.51[e]	14:45	75	9/19/14	Friday	75

[a] *n*s represent mean of two coders' estimates (where appropriate). Number rounded down if mean was a fractional value.
[b] In minutes since midnight
[c] At start of trial, data obtained from http://www.wunderground.com/history
[d] Includes two single donations of $5 and one single donation of $10
[e] Includes one single donation of $10

Table S2

Raw Means and Standard Deviations for Social Perceptions of Competence, Warmth, Self-other Similarity, and Ascribed Humanity by Condition (Study 2)

Measure	Condition	Mean	SD
**Competence**	Overall	52.65	23.10
	Low Status	47.88	22.84
	High Status	57.38	22.42
**Warmth**	Overall	57.89	23.45
	Low Status	53.99	23.91
	High Status	61.76	22.37
**Self-Other Similarity**	Overall	2.40	1.40
	Low Status	2.26	1.34
	High Status	2.54	1.46
**Ascribed Humanity**	Overall	80.10	11.41
	Low Status	78.36	12.08
	High Status	81.82	10.45

Additional Analyses for Field Experiment

Analyses accounting for city. As a follow-up analysis, we also assessed whether the effect of status signaling differed in New York and Chicago. For lack of a multifactorial non-parametric test, we report this analysis as a two-way Analysis of Variance (ANOVA), with the caveat that variances likely differ across condition. This analysis revealed that New York and Chicago pedestrians were equally generous, and city location did not moderate the effect of condition. The effect of the manipulation remained robust, $F(1, 4532) = 6.28$, $p = .01$, after controlling for the city in which the trials were conducted (M_{NY} =\$0.024, SD_{NY} = \$0.32; M_{Chi} = \$0.013, SD_{Chi} = \$0.22), $F(1, 4532) = 1.98$, $p = .16$, and the interaction between condition and city, $F(1, 4532) = 0.476$, p = .49.

Negative binomial mixed linear model approach. Given that trials were nested within location and the data contained a large number of zeros, we also performed a negative binomial generalized mixed model regressing donation (as count data) on status condition, treating location as a random intercept. As noted, we do not have individual-level data for donations, aside from those who gave in larger denominations (i.e., those of $5 or larger). Thus, we can only approximate the values that fall between $0 and $5. For the purposes of this analysis, we treated all of these unknown donations as $1. Assuming at least some linearity in the distribution of donation amounts across conditions (i.e., assuming that those in the low status condition were no more likely to give just over $1 or those in the high status condition no more likely to give just under $1 than their respective counterparts), this test likely represents a conservative test of our hypothesis because the average amount given, across participants, tended to be higher in the relatively high status condition.

As expected, accounting for the random intercept of location (*AIC* = 553.57, *BIC* = 579.24) improved the model over a null model (*AIC* = 561.48, *BIC* = 587.16), $\chi^2 = 7.91$. After accounting for the random intercept of location (*SD* = .70), exposure to the relatively high status condition remained a positive (and, indeed, a stronger) predictor of donation amount, *b* = 1.22, *z* (4532) = 3.31, *p* = .001.

Additional Analyses for Perceptual Study

We also performed sensitivity analyses by dropping those who chose "none of the above" in response to our attention check.

Again, participants rated the relatively high status confederate (*M* = 3.52, *SD* = 1.89) as higher in status than the relatively low status confederate (*M* = 2.44, *SD* = 1.68), *t*(456.99) = 6.52, *p* < .001; both of these means were, again, above the midpoint of the scale: *t*(242) = 12.16, *p* < .001 and *t*(215) = 22.467, *p* < .001, respectively.

A Multivariate Analysis of Variance (MANOVA) revealed that the social status manipulation continued to influence the hypothesized perceptions of the target in much the same manner (with the exception that the effect of condition on IOS is now marginal, likely a consequence of a loss of statistical power), Wilk's $\lambda = .96$, *F*(4,453) = 4.82, *p* < .001. Specifically, participants judged the higher status target as more competent, *F*(1,456) = 17.64, *p* < .001, *d* = 0.39 [95% CI: 0.21, 0.58], warmer, *F*(1,456) = 10.65, *p* = .001, *d* = 0.31 [95% CI: 0.12, 0.49], marginally more similar to the self, *F*(1,456) = 3.69, *p* = .055, *d* = 0.18 [95% CI: 0, 0.36], and more human, *F*(1,456) = 7.03, *p* = .008, *d* = 0.25 [95% CI: 0.06, 0.43] than the lower status one.

Table S3

Correlations among perceived social status, competence, warmth, IOS, and ascribed humanity, perceptual study (degrees of freedom in parentheses).

	1	2	3	4	5
1. Perceived Subjective SES (of target)	--	.42*** (490)	.35*** (490)	.25*** (489)	.17*** (490)
2. Competence		--	.87*** (490)	.42*** (489)	.51*** (490)
3. Warmth			--	.38*** (490)	.53*** (490)
4. IOS				--	.19*** (489)
5. Ascribed Humanity					--
